# Cytokines and Disease

**DOI:** 10.1155/2014/209041

**Published:** 2014-09-14

**Authors:** Arkadiusz Orzechowski, Agueda A. Rostagno, Sabina Pucci, Gilles Chiocchia

**Affiliations:** ^1^Department of Physiological Sciences, Warsaw University of Life Sciences (SGGW), Nowoursynowska 159, 02-776 Warsaw, Poland; ^2^Electron Microscopy Platform, Mossakowski Medical Research Centre PAS, Pawińskiego 5, 02-106 Warsaw, Poland; ^3^Department of Pathology, New York University, School of Medicine, 550 First Avenue, MSB 556, New York, NY 10016, USA; ^4^Department of Biomedicine, University of Rome “Tor Vergata”, Policlinico “Tor Vergata”, Viale Oxford, 00133 Rome, Italy; ^5^Simone Veil Department of Health Sciences, University Versailles-Saint-Quentin, 78180 Montigny-Le-Bretonneux, France

Recent evidence in biology and medicine has expanded the knowledge of cytokines function in many disease states and conditions as well as the severe adverse effects implicated in the escalated production associated with specific disease conditions. This special issue was intended to provide an updated view on the role of cytokines in the disease pathogenesis of degenerative and neoplastic disorders. Special emphasis has been dedicated to the contribution of cytokines to systemic conditions including but not limited to neurodegeneration, cancer, and graft/prosthesis rejection. To address these issues we invited authors who could shed light on the contribution of cytokines on such diseases, identify mechanisms employed by tumor cells to subvert the host cytokine response, provide new cellular and animal models to test and understand the activity of cytokines in degenerative and neoplastic diseases, highlight cytokines involved in neurodegeneration, and illustrate how cytokines affect graft and prosthesis rejection.

One of the papers of this special issue addresses the dynamics of acute local inflammatory response to the intramuscular cell transfer in autologous cell models. Early immune reaction (infiltration with neutrophils and macrophages) at the site of injection was accompanied by considerable rise of* Il-1α, Il-6, Tgf-* *β*, and* Tnf*-*α* gene expressions. Autotransplantation of muscle-derived cells into skeletal muscle resulted in marked decrease of viable transplanted cells which may contribute to the low efficacy of cellular grafts. Another paper presents the observations collected from children with allergic rhinitis (AR). The authors showed that the anti-inflammatory cytokine IL-37b, a member of the interleukin 1 cytokine family whose expression is related to the efficacy of intranasal synthetic steroid (mometasone furoate) therapy, downregulated the expression of Th2 cytokines in PBMCs through MAPK and PI3-K signaling pathways in AR. The paper entitled “*Tumour necrosis factor superfamily members in the pathogenesis of inflammatory bowel disease”* reviews the contribution of the tumor necrosis superfamily (TNFSF) to the pathogenesis of inflammatory bowel disease (IBD). Special attention was drawn on TL1A, FasL, LIGHT, TRAIL, and TWEAK as possible targets in IBD treatment through their role on apoptosis and T-cell differentiation. One of the papers of this special issue is a review featuring TNF-*α* as a proinflammatory cytokine exerting both homeostatic and pathophysiological roles in the central nervous system. It summarizes the current knowledge of the cellular and molecular mechanisms by which TNF-*α* links the neuroinflammatory and excitotoxic processes that occur in several neurodegenerative diseases, but with a special emphasis on amyotrophic lateral sclerosis (ALS). It stresses the* de novo* production of TNF-*α* by microglia as an important component of the so-called neuroinflammatory response which may represent a valuable target for intervention. The paper entitled “*Protective effects of lipoxin A4 in testis injury following testicular torsion and detorsion in rats” *describes the study on the protective effects of lipoxin A4 (LXA4), a lipid mediator with potent anti-inflammatory properties, in rat testis injury following testicular torsion/detorsion. Lipoxin A4 protective effect takes place via modulation of proinflammatory cytokines, oxidative stress, and NF-*κ*B activity. The paper by J. M. Zarzynska addresses the current view on the dual role played by TGF-*β*1 in breast cancer insurgence and progression. In early stages of breast cancer, TGF-*β*1 inhibits epithelial cell cycle progression and promotes apoptosis, showing tumor suppressive effects. However, in late stages of the disease, TGF-*β*1 cooperates to promote tumor progression by orchestrating the tumor microenvironment cytokines production, influencing tumor cell motility, invasion genes expression, cancer invasiveness, and metastasis. TGF-*β*1 is also involved in cancer microenvironment modification and promotion of epithelial to mesenchymal transition. According to current knowledge, several drugs counteracting TGF-*β*1 have been developed and are either in preclinical or in early stages of clinical investigation. One of the papers evaluates the importance of intestinal homeostasis for the healthy status of the large bowel. The authors present numerous data that substantiate dietary essential n-3 polyunsaturated fatty acids (PUFAs) and short-chain fatty acid butyrate as anti-inflammatory and anticancer agents. PUFAs modulate the production and activities of TNF family cytokines (TNF-*α*, TRAIL, and FasL) that have potent inflammatory activities. The possible application for the prevention and therapy of colon inflammation and cancer is also outlined. The final paper of this special issue outlines the effects of the antibiotic florfenicol on the expected changes in sTNF-*α*, damage markers of the liver and kidney, and the lipid metabolism parameters in endotoxemic brown trout. The paper concludes that florfenicol does not affect the LPS-mediated increases of sTNF-*α* and does not prevent liver or kidney damage indicating that the antibiotic does not have any evident positive effects on acute endotoxemia in fish.

Overall, the current issue highlights the multidisciplinary contribution of cytokines, through their broad spectrum signaling capability and regulation of cellular communication, as crucial mediators in diverse pathologic entities with broad etiology.

*Arkadiusz Orzechowski* *Arkadiusz Orzechowski* *Agueda A. Rostagno* *Agueda A. Rostagno* *Sabina Pucci* *Sabina Pucci* *Gilles Chiocchia* *Gilles Chiocchia*

